# Postpartum retention in opioid agonist treatment for opioid dependence: A population-based cohort study

**DOI:** 10.1007/s00737-025-01640-8

**Published:** 2026-01-06

**Authors:** Joanna Zhou, Bianca Varney, Nicola Jones, Chrianna Bharat, Louisa Degenhardt, Alys Havard, Duong Thuy Tran

**Affiliations:** 1 National Drug and Alcohol Research Centre, UNSW Sydney, Sydney, Australia; 2 Medicines Intelligence Research Program, School of Population Health, Faculty of Medicine and Health, UNSW Sydney, Sydney, Australia

**Keywords:** Opioid dependence, Opioid agonist treatment, Retention, Postpartum, Retrospective cohort study, Socio-demographic determinants

## Abstract

**Purpose:**

Opioid agonist treatment (OAT) is the first-line treatment for opioid dependence during pregnancy and recommended for at least one year postpartum or until a strong maternal-infant bond and stable family environment is established. Evidence on postpartum OAT retention is limited. We examined retention rates and associated maternal characteristics.

**Methods:**

We linked OAT prescription authority to perinatal, mortality, and other data sources. We identified all opioid-dependent women who gave birth in New South Wales, Australia (1 January 2004-31 March 2020) while receiving OAT. We defined retention at 90, 180, and 365 days postpartum as continuous treatment over each period. We calculated retention rates and used generalised linear modelling to examine association between retention and maternal socio-demographic and clinical factors.

**Results:**

There were 3933 childbirths among 2514 women on OAT. Retention rates were 93.3% (*n* = 3670) at 90 days, 88.4% (*n* = 3475) at 180 days, and 78.5% (*n* = 3086) at 365 days. Retention at 180 days was lower for those who gave birth after 2015, were Indigenous, had recent conviction or incarceration, initiated OAT after conception, received buprenorphine, or had a mental illness. We observed similar association patterns at 90 and 365 days.

**Conclusions:**

Among women on OAT at childbirth, postpartum treatment retention was high but varied across subgroups. Lower retention among women who initiated OAT late in pregnancy or with social or clinical risk factors highlights the need for targeted support. Lower buprenorphine retention warrants ongoing monitoring and tailored care, particularly in settings where it is the preferred treatment during pregnancy.

**Supplementary Information:**

The online version contains supplementary material available at 10.1007/s00737-025-01640-8.

## Highlights

Opioid agonist treatment (OAT) is the first-line treatment for opioid dependence during pregnancy. In New South Wales, Australia, eight in ten women who were on OAT at the time of childbirth (January 2004-March 2020), were retained in treatment one year postpartum as per clinical recommendations.

Our findings highlight the importance of targeted support for women who initiated OAT late during pregnancy and those with social or clinical risk factors associated with lower retention.

Continued monitoring is essential, given lower retention rates among women on buprenorphine, which is the preferred first-line treatment during pregnancy, and the overall decline in retention in recent years.

**Supplementary Information:**

The online version contains supplementary material available at 10.1007/s00737-025-01640-8.

## Introduction

Opioid agonist treatment (OAT) with methadone or buprenorphine is the first-line treatment for pregnant women with opioid dependence to reduce perinatal harms (New South Wales Ministry of Health, 2024, The American College of Obstetricians and Gynaecologists 2017; World Health Organization 2014). During pregnancy, OAT prevents repeated exposure to intoxication and withdrawal episodes, lowering the risks of preterm delivery, pre-eclampsia, caesarean section, low birth weight, and perinatal mortality (The American College of Obstetricians and Gynaecologists 2017; New South Wales Ministry of Health, 2024, Tobon et al. 2019). In the postpartum period, OAT supports recovery, and reduces relapse to promote parenting capacity and long-term outcomes (Schiff et al. 2021). Additionally, OAT reduces the risk of other harms including blood-borne infection, overdose, mortality, and high-risk drug-seeking activity which can expose individuals to medical, legal and social consequences (The American College of Obstetricians and Gynaecologists 2017). Clinical guidelines recommend initiating or maintaining OAT during pregnancy and delaying tapering until at least one year postpartum or until the maternal-infant bond is well established (The American College of Obstetricians and Gynaecologists 2017; New South Wales Ministry of Health, 2024).

The first year after childbirth poses unique challenges to OAT retention (Nawaz et al. 2022). In the initial six months, mothers recover from childbirth, adapt to physiological and practical changes, and establish infant feeding, often requiring OAT dosage adjustments as blood volume and metabolism decline (Pace et al. 2014). In the later half, caregiving demands increase, mothers may return to work, and access to postpartum care often decreases (Nawaz et al. 2022; Mburu et al. 2024). A Massachusetts, United States (US) study reported that the risk of opioid overdose increases as the postpartum period progresses, peaking at 7–12 months (Schiff et al. 2018). These insights highlight the need to quantify OAT retention across postpartum phases to inform OAT retention support strategies.

Despite recognition of these challenges, data on postpartum OAT retention remains limited and inconsistent (Mburu et al. 2024). Several studies, mostly conducted in the US, report that between 50.9% and 79.5% of opioid-dependent women receiving OAT during pregnancy remained in treatment within 12 months postpartum (Krans et al. 2021; Schiff et al. 2021; Lo-Ciganic et al. 2019; Ray-Griffith et al. 2021; Ellis et al. 2019; Wilder et al. 2015; Nawaz et al. 2022; O’Connor et al. 2018). Postpartum retention varied by maternal demographic characteristics, geographic location, insurance status, and treatment engagement during pregnancy (Lo-Ciganic et al. 2019). Lower retention was observed among women aged under 25 years, black non-Hispanic and Hispanic women, those using illicit drugs in late pregnancy, experiencing incarceration, or those with fewer healthcare visits (Lo-Ciganic et al. 2019; Ray-Griffith et al. 2021; Austin et al. 2023; Martin et al. 2023; O’Connor et al. 2018; Schiff et al. 2021). In contrast, OAT initiation before conception or in early pregnancy, and longer treatment duration during pregnancy were associated with greater postpartum retention (O’Connor et al. 2018; Krans et al. 2021; Lo-Ciganic et al. 2019; Schiff et al. 2021).

To date, only one study has compared postpartum retention between buprenorphine and methadone, reporting superior retention with buprenorphine (Schiff et al. 2021). This contrasts with well-established evidence in the general population where methadone has demonstrated greater retention (Degenhardt et al. 2023; Ecker et al. 2019; Burns et al. 2015). However, the study acknowledged possible underreporting of methadone claims in 2014 due to transitions from facility-based to statewide reporting systems (Schiff et al. 2021).

The US opioid crisis represents unique challenges, including a large number of affected individuals and low OAT coverage (Colledge-Frisby et al. 2023; Institute for Health Metrics and Evaluation 2025), limiting generalisability to Australia and other high-income countries. Australia has the highest OAT coverage globally (Colledge-Frisby et al. 2023); however, there is inadequate Australian data on the extent to which opioid-dependent women were retained on OAT after childbirth. To inform targeted strategies for supporting OAT engagement following childbirth, we quantified the rates of OAT retention at 90 days, 180 days and 365 days postpartum, and examined whether OAT retention varies according to maternal socio-demographic, treatment and clinical characteristics which are easily identifiable in clinical settings.

## Methods

### Data source

We conducted a retrospective population-based study for all women who gave birth (01 January 2004- 31 March 2020) in New South Wales (NSW), Australia while on OAT. NSW is the most populous Australian state, accounting for 30% of births (Australian Bureau of Statistics 2024) and home to 40% of Australians receiving OAT (Larney et al. 2017).

We used data from the Opioid Agonist Treatment and Safety II Study which linked OAT records to perinatal, hospitalisation, mental health, criminal justice, and death data. Details of data sources, linkage and cleaning have been reported elsewhere (Jones et al. 2025). The Controlled Drugs Data Collection (our extract covers January 2001 - November 2022) contained records of authority to prescribe OAT under the NSW Opioid Treatment Program, providing information about OAT type (methadone or buprenorphine, with no distinction between formulations available), and authorisation dates (start, expiry and cancellation). Authorities to prescribe are valid for 12 months and must be renewed for continuity (New South Wales Ministry of Health 2018; Jones et al. 2021). We defined an OAT episode as continuous authority to prescribe OAT, allowing for a gap ≤ 6 days. Sublingual buprenorphine formulations were available throughout the entire study period, while subsidized long-acting injectable buprenorphine became available in September 2019. We considered a new treatment episode as a break of > 6 days in prescription authorities (or > 36 days for buprenorphine in custodial settings from September 2019 onwards, reflecting the high adoption of long-acting injectable buprenorphine in these settings) (Bharat et al. 2024). For each episode, we calculated episode start and end dates.

The NSW Perinatal Data Collection captures all live births and stillbirths ≥ 20 weeks gestation or ≥ 400 g birthweight with maternal, obstetric, labour, delivery and neonatal details. For our extract (July 2001 - December 2021) only the year and month of childbirth were available, therefore we assigned childbirth to the last day of the month. We calculated the date of conception (DOC) as the date of childbirth *−* gestational age (days) *7 + 14 days (Tran et al. 2017).

The Admitted Patient Data Collection provided discharge records from public and private hospitals and day procedure centres. The Mental Health Ambulatory Data Collection contained contacts with public mental health services. In-hospital and mental health data, diagnoses were coded according to the International Statistical Classification of Diseases and Related Health Problems, Tenth Revision-Australian Modification. The Bureau of Crime Statistics and Research’s Re-offending Database provided finalised legal actions (e.g., criminal court appearances, custody entries and exits). The NSW Registry of Births, Deaths and Marriages supply the date of deaths registered in NSW. Details of data sources are in Appendix, Table S1.

### Study population

**Fig. 1:**
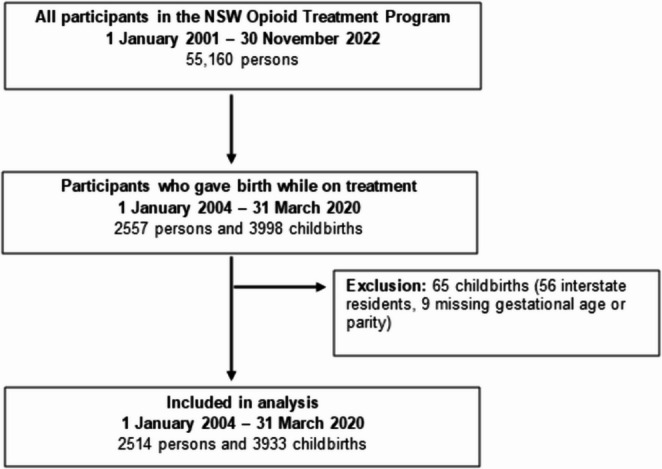
Study flow diagram

We identified a cohort of women who gave birth in NSW between 1 January 2004 and 31 March 2020 while receiving OAT (episode start date ≤ date of childbirth ≤ episode end date). We excluded interstate residents and childbirth records with missing gestational age or parity data (Fig. 1).

### Defining retention in OAT after childbirth

We defined follow-up periods of 90, 180, and 365 days from the date of childbirth, censoring at the maternal date of death or date of conception of a subsequent child, whichever occurred first. For each follow-up period, we summed all the days in which a woman received OAT. We classified a woman as retained in treatment if the entire (100%) follow-up period was covered by OAT, and classified a woman as not retained in treatment if OAT coverage was less than 100% of the follow-up period.

### Characteristics

Socio-demographic characteristics included maternal age at childbirth, country of birth, Indigenous status, recent criminal conviction or incarceration (within 365 days prior to conception or during pregnancy), remoteness of residence and socioeconomic status of area of residence. Remoteness of residence was categorised according to the Remoteness Index of Australia Plus 2021 (Australian Centre for Housing Research 2023) and socioeconomic status quintiles were defined according to the Index-of-Relative-Socio-Economic Disadvantage (Australian Bureau of Statistics 2022). Treatment characteristics included OAT type at the time of childbirth (buprenorphine or methadone), timing of OAT initiation during pregnancy (categorised as before conception, trimester 1, 2 and 3), and receipt of OAT within two years before conception. Clinical characteristics included parity, morbidities (mental health conditions involving care in hospital or publicly funded outpatient services, cigarettes smoking, an overdose event involving opioids or other substances), and birth outcomes (maternal obstetric complications, neonatal morbidity complications and neonate discharge status), with morbidities being measured in the period from 365 days prior to conception to childbirth. Detailed definitions are provided in the Appendix, Table S2.

### Statistical analysis

Analysis was conducted using SAS version 9.3 (SAS Institute, Cary, NC, USA) (SAS Institute Inc 2011). The unit of analysis was childbirth. We used descriptive statistics (frequency, proportion, mean, standard deviation [SD]) to summarise cohort characteristics and outcomes. We calculated the rates and 95% confidence intervals (CI) of women who were retained in OAT separately for the 90, 180 and 365-day follow-up periods, adjusting for multiple childbirths from the same woman, using a Huber-White robust variance estimate (Huber 1967; White 1982).

To examine the variations in treatment retention according to socio-demographic, treatment, and clinical characteristics, we calculated crude and adjusted odd ratios (OR) and 95% CI using generalised linear modelling. The multi-variable logistic regression models included year of childbirth, maternal age, country of birth, Indigenous status, incarceration or conviction, residential remoteness, residential socioeconomic status, OAT type, timing of OAT initiation, parity, mental health, overdose, and smoking. OAT use within two years pre-conception was not modelled as it was strongly correlated with timing of OAT initiation (correlation coefficient 0.56).

## Results

Between 01 Jan 2004 and 31 March 2020, there were 3,998 births among 2,557 opioid-dependent mothers receiving OAT. After exclusion of 65 childbirths (interstate residence and missing data), the analysis included a cohort of 3,933 childbirths among 2,514 women (Fig. 1).

Mean maternal age was 30.2 years (SD 5.4). The cohort included 362 childbirths (9.2%) to overseas-born women, 875 (22.2%) to Indigenous women, and 1240 (31.5%) to women with a recent incarceration or conviction. Most childbirths occurred in major cities (77.0%, *n* = 3030) and in the two most socioeconomically disadvantaged quintiles (66.3%, *n* = 2608). Most women had OAT within the two years before conception (89.1%), initiated OAT preconception (72.1%), and were on methadone at the time of birth (84.0%). Cigarettes smoking was common (90.5%) while 16.0% had a mental health condition. In 1451 childbirths (36.9%) mothers experienced an obstetric complication while neonatal complications occurred in 2488 childbirths (63.3%) (Table 1).

**Table 1:** Description of the cohort: socio-demographic characteristics, opioid agonist treatment, maternal health and birth outcomes (*N* = 3933)

	Characteristics	Number	Proportion (%)
Year of childbirth			
2004–2007		1389	35.3
2008–2011		1092	27.8
2012–2015		876	22.3
2016–2018		439	11.2
2019–2020		137	3.5
Maternal age at childbirth (years)			
Under 25		618	15.7
25–30		1470	37.4
31–35		1137	28.9
Over 35		708	18.0
Country of birth			
Australia		3557	90.4
Overseas		362	9.2
Missing		14	0.4
Indigenous status			
Non-Indigenous		3058	77.8
Indigenous		875	22.2
Incarceration or conviction ^a^			
No		2693	68.5
Yes		1240	31.5
Remoteness of residence ^b^			
Major city		3030	77.0
Inner regional		740	18.8
Outer regional, remote, very remote		163	4.1
Socioeconomic status of residence ^c^			
Quintile 1: Least disadvantaged		329	8.4
Quintile 2		354	9.0
Quintile 3		642	16.3
Quintile 4		1067	27.1
Quintile 5: Most disadvantaged		1541	39.2
Type of OAT at childbirth			
Methadone		3303	84.0
Buprenorphine		630	16.0
Timing of OAT initiation during pregnancy			
Before conception		2837	72.1
First trimester		396	10.1
Second trimester		451	11.5
Third trimester		249	6.3
OAT use within two years pre-conception			
No		427	10.9
Yes		3506	89.1
Parity			
Nulliparous		848	21.6
1 or 2		1752	44.6
3 or more		1333	33.9
Mental health conditions ^a^			
No		3303	84.0
Yes		630	16.0
Overdose involving opioids or other substances ^a^			
No		3795	96.5
Yes		138	3.5
Cigarette smoking ^a^			
No		373	9.5
Yes		3560	90.5
Maternal obstetric complications ^d^			
No		2482	63.1
Yes		1451	36.9
Neonatal morbidity complications ^e^			
No		1445	36.7
Yes		2488	63.3
Neonate discharge status			
Alive		3795	96.5
Dead or stillborn		98	2.5
Not stated		40	1.0

a Incarceration or conviction, mental health, an overdose involving opioids or other substances and cigarette smoking were measured in the period from 365 days before conception to childbirth

b Residential remoteness was categorised using the Remoteness Index of Australia Plus 2021

c Socioeconomic disadvantage quintiles were defined according to the Index-of-Relative-Socio-Economic Disadvantage, Australian Census 2011

d Maternal obstetric complications included post-partum haemorrhage requiring blood transfusion, caesarean sections, or placental abruption

e Neonatal morbidity complications included preterm births before 37 weeks, neonatal resuscitation, Apgar scores at 5 min < 7, birthweight < 2500 g, or small for gestational age (< 10th sex and gestational-age specific percentile)

### Postpartum retention in treatment

Overall, OAT retention at 90 days was 93.3% (*n* = 3670), declining to 88.4% (*n* = 3475) at 180 days, and 78.5% (*n* = 3086) at 365 days postpartum (Table 2).

**Table 2:** Descriptive statistics for treatment retention at 90-day, 180-day, and 365 days postpartum

	Postpartum period		
	90 days	180 days	365 days
Total cohort at baseline	3933	3933	3933
Maternal death or subsequent conception during the postpartum period (frequency)	33	181	482
Person-days of follow-up ^a^			
Mean (days, SD)	89.9 (2.0)	177.5 (13.7)	346.8 (57.1)
Median (days, IQR)	90 (90–90)	180 (180–180)	365 (365–365)
Days covered by treatment ^b^			
Mean (days, SD)	87.1 (12.6)	167.9 (34.1)	314.8 (92.5)
Median (days, IQR)	90 (90–90)	180 (180–180)	365 (307–365)
Treatment retention			
Retained in treatment (frequency) ^c^	3670	3475	3086
Retention rate (95% CI) ^d^	93.3% (92.5–94.1)	88.4% (87.3–89.4)	78.5% (77.1–79.8)

*SD* standard deviation, *IQR* Interquartile range, *CI* confidence interval

^a^ Person-days of follow-up were measured from the date of childbirth, censoring at the maternal date of death or subsequent conception, whichever occurred first

^b^ During the follow-up (with censoring), days on which a woman received opioid agonist treatment were summed up

^c ^Retained in treatment was defined as all person-days of follow-up were covered by treatment

^d^ Retention rate was calculated as the number of women retained in treatment divided by the total cohort at baseline (n = 3933)

Retention in treatment at 180 days (Table 3) was lower for women on buprenorphine than those on methadone (84.8% vs. 89.0%, aOR 0.75, 95%CI: 0.57–0.98). Retention also declined with later OAT initiation: first trimester (85.4%, aOR 0.53, 95%CI 0.38–0.73), second trimester (76.9%, aOR 0.30, 95%CI 0.23–0.39) and third trimester (66.3%, aOR: 0.17, 95%CI 0.12–0.23) compared to preconception initiation (92.5%). Retention was also lower among Indigenous women compared to non-Indigenous women (83.3% vs. 89.8%, aOR: 0.66, 95% CI: 0.52–0.84), women with a recent incarceration or conviction (85.3% vs. 89.8%, aOR: 0.75, 95% CI: 0.61–0.93), and women with a mental health condition (84.4% vs. 89.1%, aOR 0.71, 95%CI 0.55–0.92).

**Table 3.**
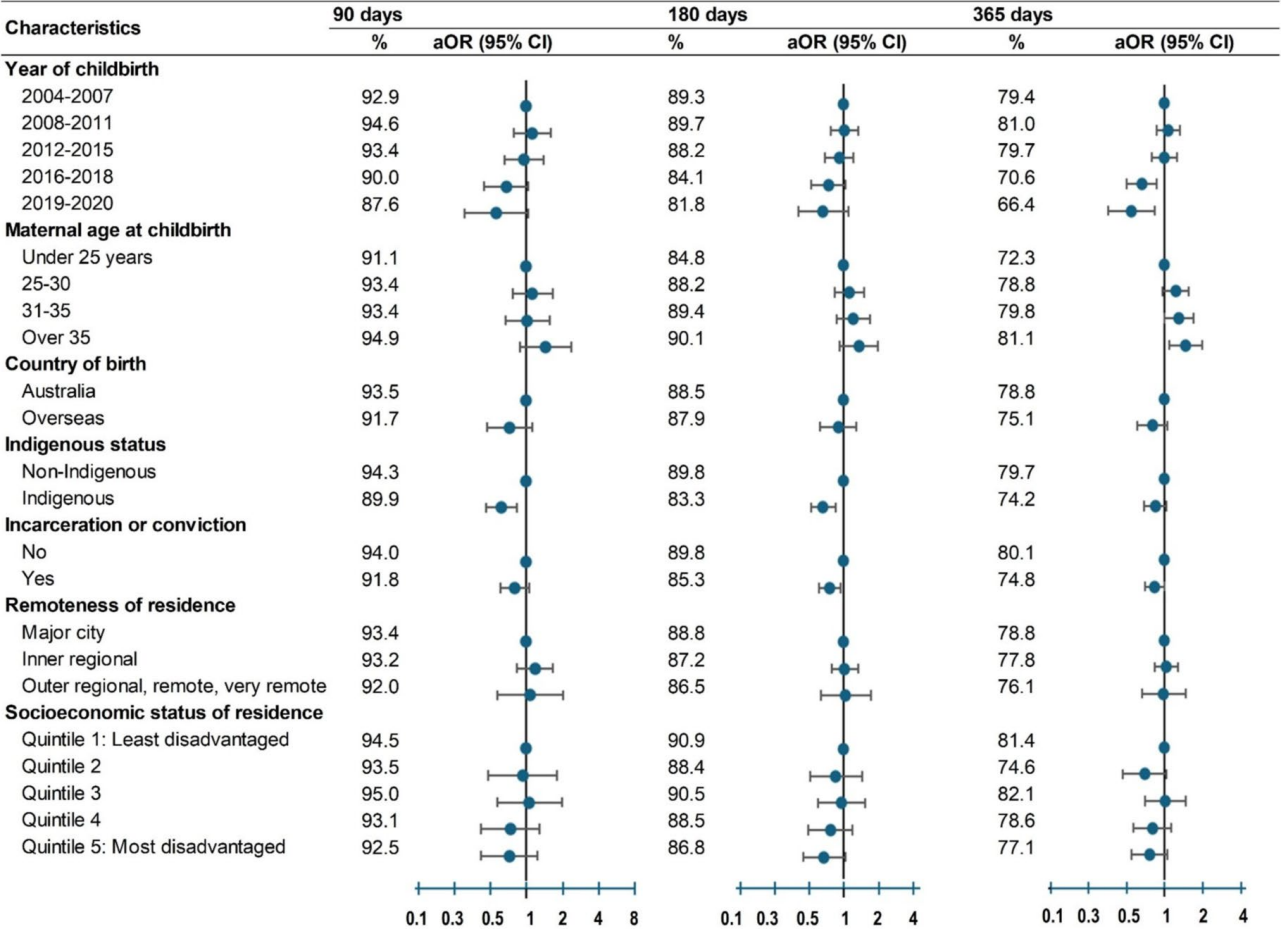

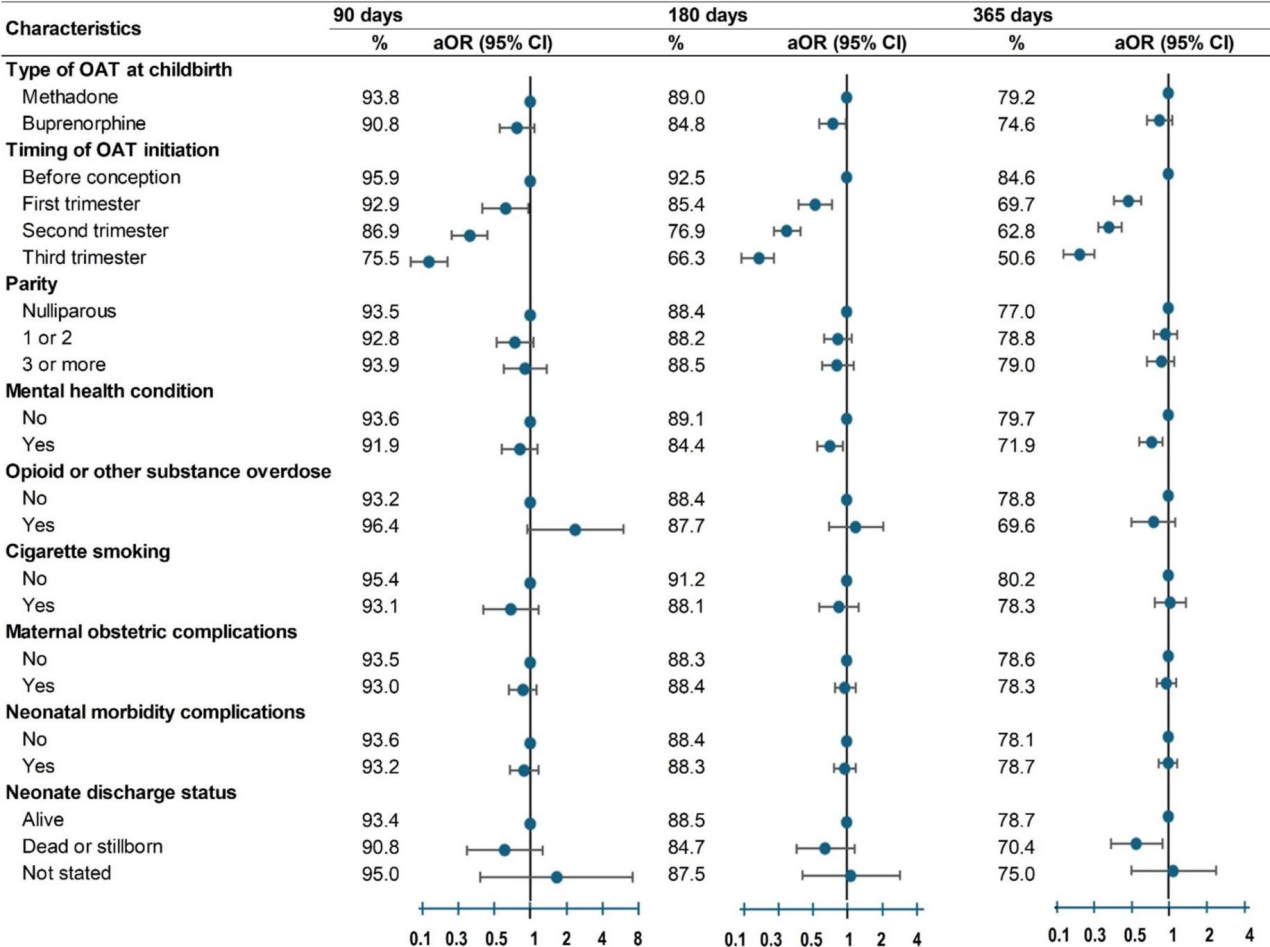
Treatment retention at 90-, 180-, and 365-days postpartum by socio-demographic and clinical characteristics among 3933 births: rate (%) and adjusted odds ratios (95%CI)

* aOR *Adjusted odds ratio, *CI* Confidence interval, *OAT* Opioid agonist treatment

^a^ Odds ratios were adjusted for year of childbirth, maternal age, country of birth, Indigenous status, incarceration or conviction, remoteness, residential socioeconomic status, OAT type, timing of OAT initiation, parity, mental health, overdose, and smoking. See Appendix, Table S2 for definitions of socio-demographic and clinical characteristics; Tables S3-S5 for number retained, rates, crude and adjusted odds ratios

Retention patterns at 90-days and 365 days (Table 3) were largely consistent with 180-day findings. Exceptions occurred for OAT type, i.e. retention for buprenorphine not statistically significant at 90 days or 365 days. At 365 days, retention was lower among mothers who gave birth in 2016–2018 (aOR:0.66, 95%CI 0.50–0.86), in 2019–2020 (aOR:0.55, 95%CI 0.36–0.83), and those whose babies stillborn or died before discharge (aOR:0.56, 95%CI 0.35–0.89). See Appendix, Tables S3-S5 for number retained, rates, crude and adjusted ORs.

## Discussion

This population-based study found that high proportions of opioid-dependent women treated with OAT at childbirth in NSW, Australia were retained in treatment in the year after childbirth. However, retention gradually declined over time from birth, from 93.3% at 90 days to 78.5% at 365 days. In the absence of data on reasons for OAT discontinuation, these decreasing retention rates may reflect planned tapering, disengagement from care or other factors.

Despite the overall high retention rates, our study identified several groups who were less likely to be retained in treatment. Retention rates at 180 days were significantly lower among Indigenous women, those with a conviction or incarceration in previous 12 months, women who initiated OAT after conception, women receiving buprenorphine, and women with a mental health condition. In addition, retention rates at 365 days were significantly lower among women who gave birth later in the study period (2016–2020), and among those who experienced a stillbirth or neonatal death. These findings underscore the need for enhanced monitoring and coordinated care throughout the first year postpartum, particularly during the second six months, to support women with these characteristics in maintaining treatment.

Compared to US studies, OAT retention rates in our study were higher at all three measured time points: 90 days (93.3% versus 50.9–73.1%), (Krans et al. 2021; Lo-Ciganic et al. 2019; Ray-Griffith et al. 2021; Ellis et al. 2019), 180 days (88.4% versus 44.0%−74.5%) (O’Connor et al. 2018; Wilder et al. 2015) and 365 days (78.5% versus 64.1%−70.9%) (Schiff et al. 2021; O’Connor et al. 2018). This may be partly attributable to the accessibility of services such as the NSW Substance Use in Pregnancy Program Services which provides a case manager to support opioid-dependent women during pregnancy and after childbirth (New South Wales Ministry of Health, 2024). The case manager devises a holistic management plan, reviews treatment progress and coordinates regular multidisciplinary team meetings and referrals to necessary services (Coupland et al. 2021; Royal Hospital for Women 2024). With strong harm minimisation strategies, coupled universal health insurance (Pharmaceutical Benefits Scheme 2025), Australia has the highest OAT coverage globally, i.e. 78.4% among people who inject primarily opioids compared to the global average of 21.5% (Nicholas 2022; Colledge-Frisby et al. 2023). Additionally, Australia has a robust network of non-government organisations e.g. the Network of Alcohol and other Drugs Agencies (Network of Alcohol and Other Drugs Agencies, 2025), and peer-led peak organisation e.g. the Australian Injecting and Illicit Drug Users League and its state-based member organisations (Australian Injecting and Illicit Drug Users League 2025) to support people who use substances. Initiatives co-developed with or delivered by community-led organisations have reduced stigma among people attending health services for alcohol and drug-related reasons (Conway A., 2022).

Nevertheless, differences in retention rates between our study and prior studies may also reflect variations in study populations and definitions of retention. Although our study included all women in NSW who were treated with OAT at childbirth, most US studies focused only on women enrolled in specialised treatment programs involving structured visits and regular urine toxicology screens (Ray-Griffith et al. 2021). Additionally, our study defined retention as continuous OAT coverage for the entire postpartum period, whereas US studies varied in definitions, such as having at least one claim, attending scheduled appointments, or receiving treatments without a gap of two consecutive months (Mburu et al. 2024; Krans et al. 2021; Ray-Griffith et al. 2021).

In this study, postpartum retention was lower among women receiving buprenorphine compared with those on methadone. This aligns with the pharmacological property of buprenorphine, a partial agonist, that is generally less efficacious than methadone, a full agonist, in controlling withdrawal and cravings, particularly among individuals with severe opioid dependence (Degenhardt et al. 2023; Krans et al. 2016). Our findings corroborate prior evidence from general and perinatal populations showing greater dissatisfaction and lower retention in buprenorphine compared with methadone (Ecker et al. 2019; Burns et al. 2007, 2015; Degenhardt et al. 2023). The recent introduction of long-acting injectable buprenorphine formulations (Bharat et al. 2024) may offer the potential to improve retention by reducing the burden of daily dosing. However, evidence regarding the safety and effectiveness of long-acting injectable buprenorphine during pregnancy is scarce. Studies of other buprenorphine formulations, mostly sublingual buprenorphine, suggest possible superiority of buprenorphine over methadone (e.g. higher birth weight, longer gestation) (Suarez et al. 2022; Zedler et al. 2016; Kinsella et al. 2022) but these findings are subject to bias from methodological weaknesses and possible unmeasured confounding (Kinsella et al. 2022). Current Australian guidelines recommend buprenorphine as the first-line treatment for pregnant women who are not already on OAT (New South Wales Ministry of Health, 2024), and recent population-based study of the same data source (Tran, 2025) indicated a gradual increase over time in buprenorphine use during pregnancy and a steady decrease in methadone use among opioid-dependent pregnant women who had a history of OAT in NSW. These evolving trends highlight the importance of rigorous evaluation of maternal and neonatal outcomes associated with buprenorphine, including long-acting buprenorphine, and underscore the need for individualised treatment planning and shared decision-making in selecting a medicine that optimally balances maternal stability, treatment retention and health outcomes.

This study confirms prior US studies showing lower postpartum retention among women who initiated OAT during pregnancy, especially those who initiated in the second or third trimester (Schiff et al. 2021; Lo-Ciganic et al. 2019; Ray-Griffith et al. 2021; Wilder et al. 2015; O’Connor et al. 2018). Women who engage in treatment earlier may have more stable social and financial circumstances, better psychosocial and physical health, and stronger rapport with healthcare providers (Krans et al. 2021). In contrast, women who initiated OAT later in pregnancy may face additional challenges that affect treatment engagement.

Mental health was another important factor associated with lower retention in postpartum period. We found reduced retention among women who were admitted to hospital for mental illness-related reasons or sought treatment in public outpatient mental health services. Lack of mental health supports has been identified as one of the top three barriers to accessing OAT services (Hall et al. 2023). A prior study reported that women receiving antidepressant medication in the third trimester had longer retention on OAT (O’Connor et al. 2018). These insights emphasize the importance of adequate mental health support during pregnancy and after childbirth to ensure mental wellbeing and improve OAT retention in the postpartum period.

In addition, we observed lower retention rates at 365 days among women who gave birth later during our study period, 2016–2018 (70.6%), and 2019–2020 (66.4%). Despite increased availability of take-away dosing, long-acting buprenorphine and telehealth monitoring, the COVID-19 pandemic may have had inevitable impacts on OAT access and postpartum retention (Bharat et al. 2024). Online surveys and semi-structured interviews of Australian women who gave birth after March 2020 consistently reported inadequate access to postnatal care and support due to COVID lockdown restrictions and frequent cancellations of postpartum health checks (Sweet et al. 2022; Wilson et al. 2022; Grech et al. 2024). The decline in postpartum treatment retention observed between 2016 and 2020 may reflect two interrelated factors, namely the lower retention associated with buprenorphine and the growing preference for buprenorphine over methadone among opioid-dependent pregnant women with a history of OAT in NSW (Tran, 2025).

### Strengths and limitations

This is the first study reporting retention in OAT among Australian women following childbirth, over 17-years. Limitations include lacking data on the severity of opioid dependence, OAT dose or reasons for discontinuation (planned or unplanned). Mental health conditions were derived from records of inpatient or publicly funded outpatient mental health services, reflecting individuals with more complex mental health care needs. Because the OAT data did not distinguish buprenorphine formulations, only women receiving buprenorphine in custodial settings from September 2019 were considered in continuous treatment if gaps were ≤ 36 days, potentially underestimating days in treatment and retention for women receiving monthly injectable buprenorphine in the community.

## Conclusion

This study shows that most opioid-dependent women treated with OAT at the time of childbirth in NSW continued OAT through the first postpartum year. The lower rates of retention among certain subpopulations—including Indigenous women, women on buprenorphine, women who initiated OAT late in pregnancy, women with a recent incarceration or conviction, and women with complex mental health illness— highlights the need for continual monitoring and targeted support to improve treatment engagement in these populations. The reduced retention among women on buprenorphine underscore the importance of informed decision-making when selecting medication and formulation. Future studies should investigate mechanisms underlying lower retention with buprenorphine and evaluate integrated approaches to optimise long-term engagement in treatment for opioid dependence.

## Supplementary Information

Below is the link to the electronic supplementary material.

Supplementary Material 1 (DOCX 340 KB)

## Data Availability

The data underlying this article cannot be shared publicly due to the guidelines from the data custodians. To ensure privacy and confidentiality, the approval process for linking health data in NSW is governed by strict conditions concerning data storage, retention, and usage. Currently, data can be stored at a single location—UNSW Sydney—for a maximum of 7 years after publication of the results.We welcome inquiries from interested parties regarding potential secondary data analyses. It is important to note that legislation mandates that data must be stored and analysed exclusively within NSW. Requests for data access should be directed to Principal Investigator of the OATS II Study and they will be reviewed by the OATS II investigator team. Collaborators will need to obtain approval for data access and any specific secondary analyses from the NSW Population and Health Services Research Ethics Committee. Furthermore, those who are aiming to address research questions related to Aboriginal peoples must also seek approval from the Aboriginal Health and Medical Research Council.
