# Supplementary material for: Postpartum retention in opioid agonist treatment for opioid dependence: A population-based cohort study
Source: Arch Womens Ment Health. 2026 Jan 6;29(1):6. doi: 10.1007/s00737-025-01640-8 (PMC12769642; doi:10.1007/s00737-025-01640-8)
Supplement: Supplementary file 1 — Supplementary Material 1 (DOCX 340 KB) [file 737_2025_1640_MOESM1_ESM.docx]

# **APPENDIX**

## **Abbreviations**

APDC: Admitted Patient Data Collection

CODDAC: Controlled Drugs Data Collection

ICD-10-AM: International Classification of Diseases, Version 10, Australian

Modification

MH-AMB: Mental Health and Ambulatory Data Collection

NSW: New South Wales

OAT: Opioid agonist treatment

PDC: Perinatal Data Collection

RBDM: Registry of Births, Deaths and Marriages

ROD: Bureau of Crime Statistics and Research's Re-offending Data

Collection

## **Table S1: Information on data sources used in this study**

| **Data collection** | **Brief description** |
| --- | --- |
| Controlled Drugs Data Collection (CODDAC, referred to as *OAT data*) | Records of authority to prescribe opioid agonist treatment (OAT) for opioid dependence under the NSW Opioid Treatment Program. The OAT data provide Information about medication type (methadone or buprenorphine, with no distinguishable buprenorphine formulations), authority dates (start, expiry, cancellation), dosing dates (start, end), dosing setting (clinic, community pharmacy, Justice Health, and others), and the date of treatment program exit |
| Perinatal Data Collection (PDC, referred to as *perinatal data*) | A statuary, population-based data collection that contains records of all births (live or still birth) in NSW of at least 400 g birth weight or a gestational age of at least 20 weeks' gestation. The PDC includes maternal socio-demographic, medical and obstetric information, and characteristics of mothers and their infants collected at the time of birth |
| Admitted Patient Data Collection (APDC) | Statutory data collection containing records of admission and discharge for all public, private, psychiatric, and repatriation hospitals in NSW. Diagnoses are coded according to the International Classification of Diseases, Version 10, Australian Modification (ICD-10-AM) and procedures coded according to the Australian Classification of Health Interventions. |
| Mental Health and Ambulatory Data Collection (MH-AMH) | Records of contacts with NSW Ministry of Health funded mental health services for non-admitted patients; includes contacts for patient assessment, treatment, rehabilitation or care. Care can be provided though mental health day programmes, psychiatric outpatients and outreach services. |
| Bureau of Crime Statistics and Research's Re-offending Database (ROD) | Records of finalised legal actions within the NSW Criminal Justice System (e.g., criminal court appearances, juvenile cautions, youth justice conferences, custody entries and exits). |
| NSW Registry of Births, Deaths and Marriages (RBDM) | Records of death registered in New South Wales, providing information regarding date of death |

## **Table S2: Definitions of variables profiling socio-demographic and clinical characteristics**

| **Socio-demographic and clinical characteristics** | **Data sources** | **Items or ICD-10-AM codes in the lookback period (365 days prior to the estimated date of conception to date of childbirth)** |
| --- | --- | --- |
| Year of childbirth | PDC | Categorised as 2004-2007, 2008-2011, 2012-2015, 2016-2018, and 2019-2020 |
| Maternal age at childbirth | PDC | Categorised as under 25, 25-30, 31-35, and over 35 |
| Country of birth | PDC | Categorised as Australian born or overseas born |
| Indigenous status | All data collections in the Opioid Agonist Treatment and Safety II Study | Categorised as Indigenous and non-Indigenous |
| Any conviction or incarceration | ROD | Date of conviction, date of incarceration within the lookback period |
| Remoteness of residence | PDC | Statistical Area Level 2 of residence mapped to remoteness categories according to the Australian Statistical Geography Standard 2016 classification |
| Socioeconomic status quintile of residence | PDC | Statistical Area Level 2 of residence mapped to quintiles of the Index of Relative Socioeconomic Disadvantage, Census 2011. |
| Type of OAT | CODDAC | buprenorphine or methadone at the time of childbirth |
| Timing of OAT initiation during pregnancy | CODDAC  PDC | The timing of OAT initiation was based on the date of the OAT episode during which childbirth occurred, and categorized as: before date of conception (DOC), first trimester (DOC to DOC+84), second trimester (DOC+85 to DOC+189) and third trimester (DOC+190 to childbirth) |
| Receipt of OAT within the two years before conception | CODDAC  PDC |  |
| Mental health condition | APDC  MH-AMB | ICD-10-AM codes recorded within the lookback period, including the following:  Anxiety or depression:  F40, F41, F43.22, F43.23  F20.4, F31.3-F31.5, F32, F33, F34.1, F41.2, F43.2  Severe mental health disorder (schizophrenia, psychotic disorder, mood disorder, bipolar):  F20 - F25, F28 - F31, F34, F38, F39, F44, F48 |
| Overdose event involving opioids or other substances | APDC  MH-AMH | ICD-10-AM codes recorded within the lookback period  T40.0-T40.4, T40.6  X40-X45, X60-X65, Y10-Y15, T40.5, T40.8, T40.9, T43.6. |
| Cigarette smoking | PDC | Prior to 2011: smoking anytime duration pregnancy  2011 onwards: Smoking in the first half, in the second half of pregnancy |
|  | APDC | ICD-10-AM recorded in the lookback period:  Z72.0 |
|  | CODDAC | Nicotine use reported as another drug of concern in the OAT authority records occurred in the lookback period. |
| Maternal obstetric complications | PDC | Post-partum haemorrhage requiring blood transfusion, Caesarean section. |
|  | APDC | Premature rupture of membranes  ICD-10-AM code: O42 recorded in admission records occurred between estimated date of conception and childbirth.  Placental abruption  ICD-10-AM code: O45 recorded in admission records occurred between estimated date of conception and childbirth. |
| Neonatal morbidity complications | PDC | Preterm births: gestation age <37weeks  Resuscitation  Low Birthweight: birthweight < 2500gram  Small for gestational age: birthweight < 10^th^ national percentiles of birthweights (singleton, specific for sex and gestational age)  Apgar score at 5 minutes < 7 |
| Neonate discharge status | PDC | Based on variable “discharge status” |

## **Table S3: Treatment retention at 90 days postpartum, according to socio-demographic, treatment, and clinical characteristics: number (%), crude and adjusted odds ratios (95%CI)**

## **Table S4: Treatment retention at 180 days postpartum, according to socio-demographic, treatment, and clinical characteristics: number (%), crude and adjusted odds ratios (95%CI)**

## **Table S5: Treatment retention at 365 days postpartum, according to socio-demographic, treatment, and clinical characteristics: number (%), crude and adjusted odds ratios (95%CI)**
